# Unveiling research hotspots: a bibliometric study on macrophages in musculoskeletal diseases

**DOI:** 10.3389/fimmu.2025.1519321

**Published:** 2025-04-28

**Authors:** Yaohang Yue, Siyang Cao, Fuyang Cao, Yihao Wei, Aikang Li, Deli Wang, Peng Liu, Hui Zeng, Jianjing Lin

**Affiliations:** ^1^ Department of Bone & Joint Surgery, National & Local Joint Engineering Research Center of Orthopaedic Biomaterials, Shenzhen Key Laboratory of Orthopaedic Diseases and Biomaterials Research, Peking University Shenzhen Hospital, Shenzhen, China; ^2^ Shandong Second Medical University, Clinical Medical College, Weifang, China; ^3^ Department of Orthopedics, Second Hospital of Shanxi Medical University, Taiyuan, Shanxi, China; ^4^ Department of Rehabilitation Sciences, The Hong Kong Polytechnic University, Hong Kong, Hong Kong SAR, China; ^5^ Shenzhen Second People’s Hospital, The First Affiliated Hospital of Shenzhen University, Shenzhen, China; ^6^ Department of Sports Medicine and Rehabilitation, Peking University Shenzhen Hospital, Shenzhen, China

**Keywords:** macrophage, musculoskeletal diseases, bibliometrics, orthopaedics, data visualization

## Abstract

Research on the role of macrophages in musculoskeletal (MSK) diseases has significantly increased in recent years. However, a thorough evaluation of the developmental trajectory of this field, including the contributions of prominent authors and primary research themes, remains insufficient. Furthermore, the identification of emerging research hotspots requires more detailed exploration. This study collated articles and reviews addressing “macrophages in MSK diseases” published between 2004 and 2023, with all data extracted from the Web of Science database. The collected data were analyzed using a variety of bibliometric and visualization tools, such as VOSviewer, CiteSpace, GraphPad Prism, and R packages. Results indicate that China and the United States are the leading contributors in this research domain. Among the many academic institutions involved, Shanghai Jiao Tong University and the University of California stand out as the most productive. The journal “Frontiers in Immunology” had the highest publication output on this topic. The five most frequently explored research domains include Immunology, Rheumatology, Pharmacology and Pharmacy, Cell Biology, and Biochemistry and Molecular Biology. These results offer a comprehensive overview of the current state of research in this field and provide meaningful insights for guiding future studies.

## 1 Introduction

With the continuous extension of human life expectancy and the consequent aging population, the global prevalence of musculoskeletal (MSK) conditions, such as osteoporosis, osteoarthritis, and osteosarcoma, has markedly risen. This trend presents significant challenges to public health systems worldwide (1). Among older adults, the growing incidence of MSK disorders imposes a heavy burden on both individuals and society (2). In developing nations, the situation may become further exacerbated by the global economic recession and rising uncertainty. Consequently, it is imperative to clarify the pathophysiological mechanisms underlying MSK tissue repair to enhance the prognosis and therapeutic strategies for MSK diseases.

In recent years, research on the involvement of macrophages in MSK disorders has advanced considerably. Beyond their well-known roles in immune responses, macrophages have been found to fulfill a range of critical functions related to bone and joint health as well as disease progression. Studies indicate that, particularly in conditions like arthritis, macrophages do more than just clear cellular debris; they act as pivotal regulators of inflammation, bone remodeling, and tissue repair processes (3). Furthermore, synovial macrophages within joints demonstrate significant functional diversity and are increasingly recognized as vital contributors to the regulation of joint health and pathology (4). With the growing depth of understanding of macrophage biology, research is now concentrating on elucidating their precise roles in MSK diseases. For instance, the involvement of macrophages in bone injury and regeneration, along with their interactions with bone cells that modulate immune responses, has emerged as a key area of focus (5). These insights are not only unveiling new therapeutic targets but also enriching the understanding of the complex mechanisms underlying MSK diseases. In summary, the role of macrophages in MSK disorders is being redefined, and future investigations will continue to reveal their multifaceted functions in both health and disease, providing a theoretical basis for developing innovative therapeutic approaches.

Electronic documents are now widely acknowledged as indispensable resources for scientific researchers. However, without employing effective reading and analytical strategies, the sheer volume of available literature can become overwhelming, complicating the extraction of relevant insights. Bibliometric analysis, a quantitative and systematic approach to literature evaluation, offers a powerful solution to this problem (6). By leveraging literature visualization tools, researchers focusing on macrophages within the realm of MSK diseases can swiftly identify field development trends, recognize key authors and leading research institutions, and enhance the efficiency of their research endeavors.

While prior reviews have explored the role of macrophages in MSK diseases from various angles, there remains a notable absence of visual analyses summarizing research trends, key contributors, and central research themes in this domain. Hence, the primary objective of this study is to perform a bibliometric and visual analysis of macrophage-related research in MSK diseases over the past two decades, offering new perspectives for both foundational and applied research in the field. This study provides a valuable resource for both seasoned professionals and newcomers, presenting a comprehensive overview of the field’s scope, highlighting emerging areas of interest, and utilizing visual tools to develop well-informed strategies for future investigation. This approach significantly improves researchers’ efficiency and productivity. To our knowledge, no prior bibliometric analysis has specifically focused on this topic.

## 2 Materials and methods

### 2.1 Data source and search strategy

The Web of Science Core Collection (WoSCC) database, developed by Clarivate Analytics, is widely acknowledged as a premier and comprehensive platform, encompassing over 12,000 international academic journals (7). Due to its ability to deliver standardized and high-quality academic publication data, it is commonly employed for bibliometric analyses on the progression of scientific research topics (6). In this study, an extensive online search was conducted within the WoSCC database, targeting research articles and reviews concerning the role of macrophages in musculoskeletal (MSK) diseases. The search was limited to publications from January 1, 2004, to December 31, 2023. The search strategy employed a combination of Medical Subject Heading (MeSH) terms and keywords. Three authors (YHY, SYC, FYC) iteratively tested and refined the search methodology to ensure both accuracy and precision. A detailed description of the search strategy is provided in the Supplementary Materials.

### 2.2 Data collection and qualified standards

Detailed information on countries and regions within the WoSCC was refined through a systematic search and indexing process. Data related to the identified publications—including authors, titles, nationalities, publication years, affiliations, abstracts, keywords, and journal names—were compiled and made available for download in various formats.

The study followed specific inclusion and exclusion criteria. The inclusion criteria involved studies focused on macrophages in musculoskeletal diseases, with only original research articles and reviews published in English-language journals considered. Exclusion criteria included conference abstracts, commentaries, editorials, letters, and papers from journals with similar or unrelated titles. Any inconsistencies in study selection were resolved through discussion, with a third researcher (JJL) providing adjudication when necessary. In cases of unresolved disagreement, a senior orthopaedic specialist (HZ) made the final decision. The collected data were cleaned, analyzed, and summarized by all collaborators using GraphPad Prism 8 and Origin 2021.

### 2.3 Bibliometric analysis and visualization

WoSCC enables direct evaluation of the publication volume and citation frequency of individual works. The Relative Research Interest (RRI) metric is employed to aggregate and represent research activity in a particular field. RRI measures the proportion of publications within a specific research area in a given year, relative to the total publications produced that year (8, 9). This study analyzed publication data from 2004 to 2024. Data analysis was conducted using GraphPad Prism 8, while the creation of a world map was accomplished with R software, utilizing libraries such as scipy, matplotlib, numpy, and python. WoS and GraphPad Prism 8 were also employed to retrieve and analyze publication data from leading countries. Additionally, the H-index was used to evaluate the impact of a scholar’s research contributions, representing the number of publications that have been cited at least as many times as the number of those publications (10). GraphPad Prism 8 was applied to analyze the H-index, along with highly cited journals, authors, institutions, and research trends.

This research utilized VOSviewer software to construct a literature network and perform a detailed analysis of co-citation, co-occurrence, and coupling relationships among references. The R programming language was employed to improve the visualization of publications and their interrelations across various countries. Additionally, CiteSpace (version 6.1.R2) was used to identify leading journals exhibiting citation bursts, detect significant bursts in keywords and references, and conduct cluster analysis on keyword co-citation networks.

## 3 Results

### 3.1 Literature review of global perspectives

**Figure 1:**
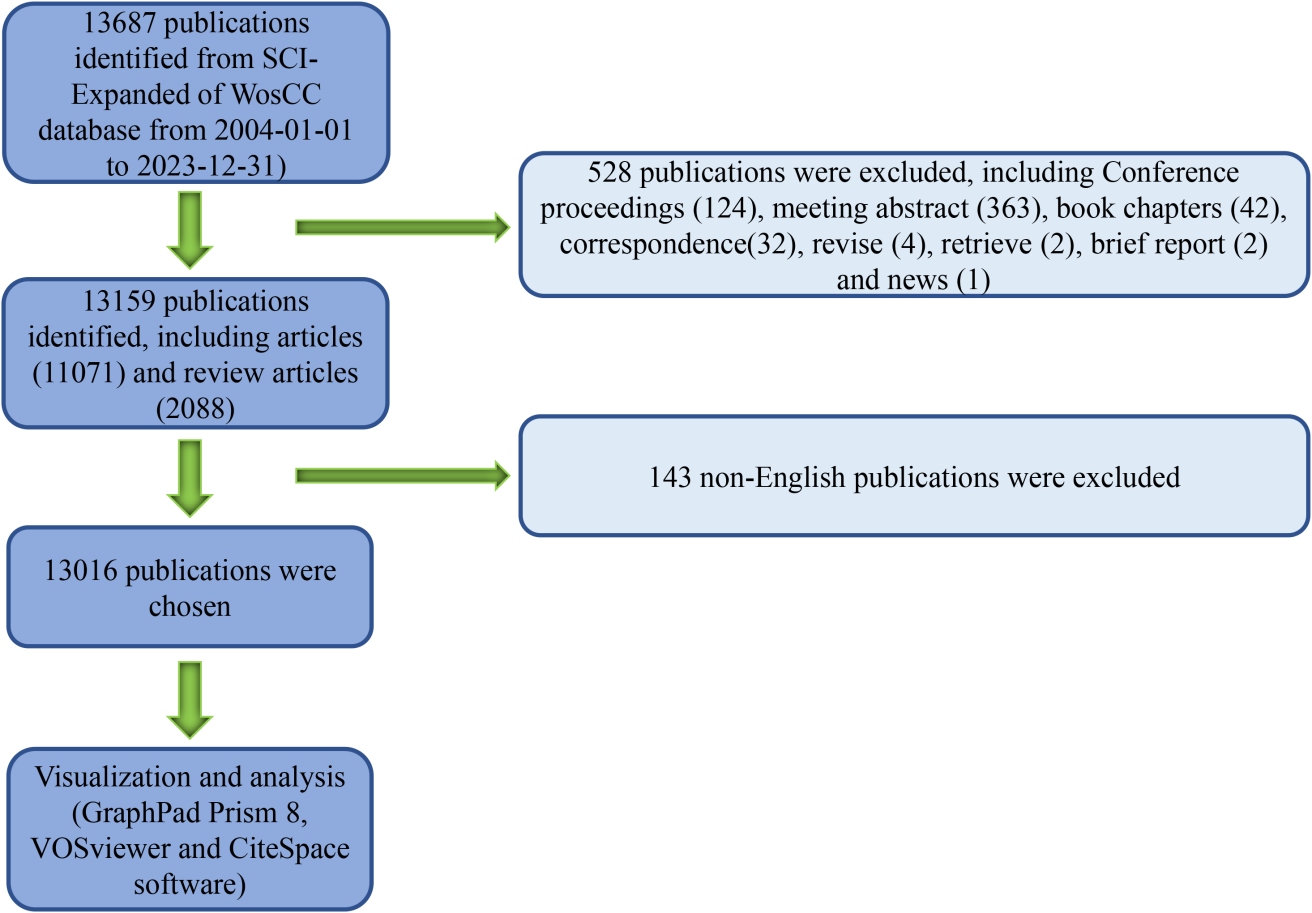
Schematic representation outlining the methodology employed for literature search and selection.

**Figure 2:**
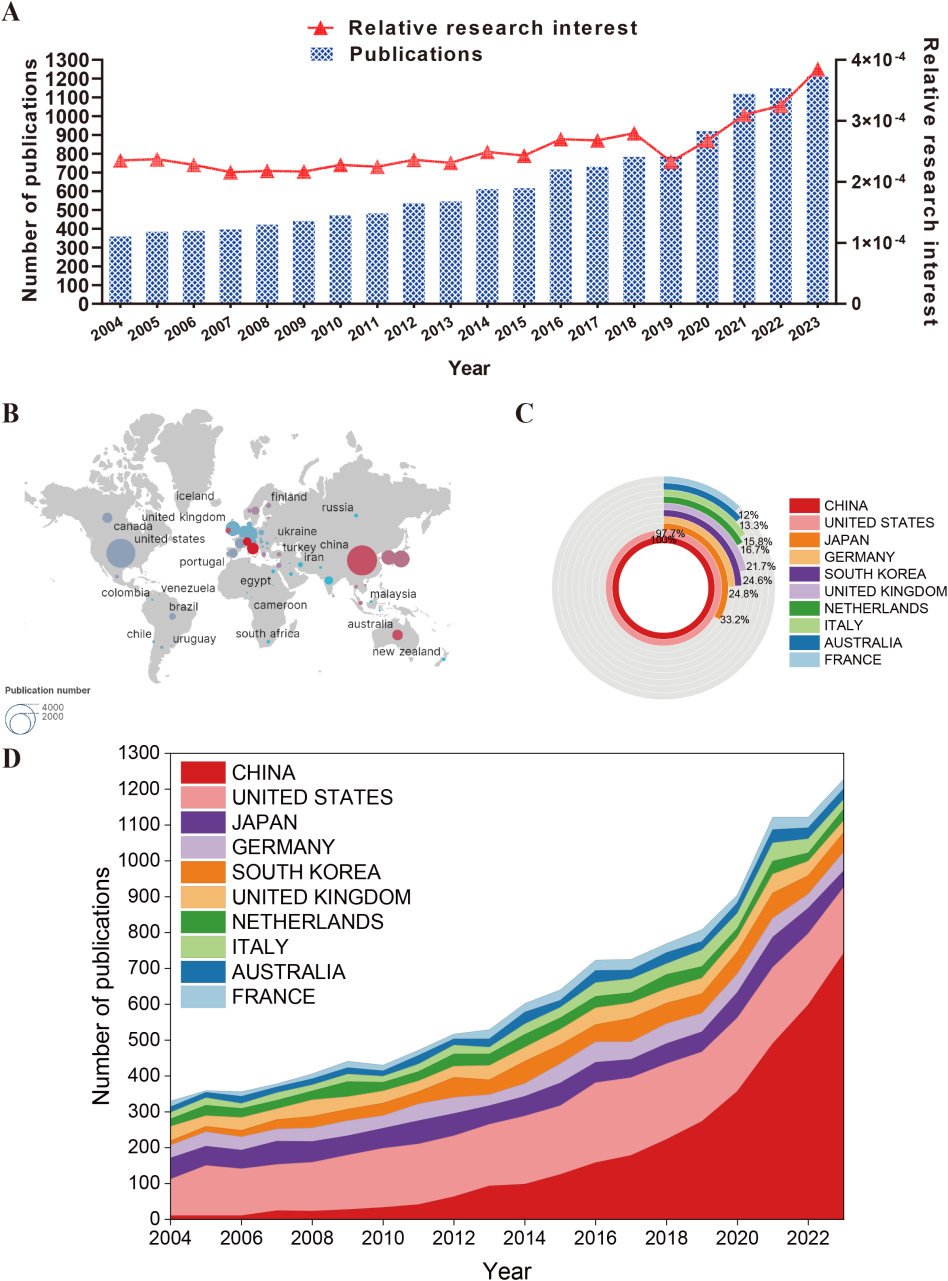
General trend of related publication worldwide from 2015 to 2022. **(A)** The trend of RRI and number of publications over time. **(B)** The distribution of publications among countries. **(C)** The top 10 countries in the field and the proportion of different countries relative to China. **(D)** An alluvium plot of the number of publications in the top 10 countries over time. The area size represents the number of publications, while the slope of the line segment represents the growth rate of publications.

Using the aforementioned methodology, a total of 13,687 relevant publications were collected spanning the years 2004 to 2023. After excluding 124 conference proceedings, 363 meeting abstracts, 42 book chapters, 32 correspondence pieces, 4 revisions, 2 retrievals, 2 brief reports, and 1 news article, the refined total amounted to 13,159 publications, including 11,071 articles and 2,088 reviews. Following the removal of 143 non-English publications, the final count reached 13,016 publications (**Figure 1**). **Figure 2A** depicts the fluctuating trend of global publication volume, which has generally increased annually, especially since 2020. There has been a steady rise in the number of papers published each year, surpassing 800, indicating a growing interest among researchers in the role of macrophages in musculoskeletal diseases as research on this topic deepens. The data illustrated in **Figure 2A** suggests significant advancements in this field of study in the near future. According to the information presented in **Figure 2B**, the primary contributors to publications over the past two decades include the countries or regions listed, with China leading, followed by the United States, Japan, Germany, South Korea, and the United Kingdom, as shown in **Figure 2C**. The trend in **Figure 2D** highlights a substantial increase in annual article output from the top 10 countries/regions, escalating from 358 in 2004 to 1,212 in 2023. By the end of 2023, China had produced a total of 3,577 papers, accounting for 27.387% of the global total and securing the top position worldwide.

The increase in scholarly publications signifies a marked interest among researchers in exploring the role of macrophages in musculoskeletal diseases. This trend highlights the emergence of a burgeoning field that is attracting greater attention from the academic community, underscoring its increasing importance. The anticipated growth in annual publications indicates that the field is likely to continue expanding, offering potential for innovative advancements in treatment strategies, understanding disease progression, and refining diagnostic criteria for musculoskeletal disorders.

### 3.2 Evaluation of publications from various countries and regions

**Figure 3:**
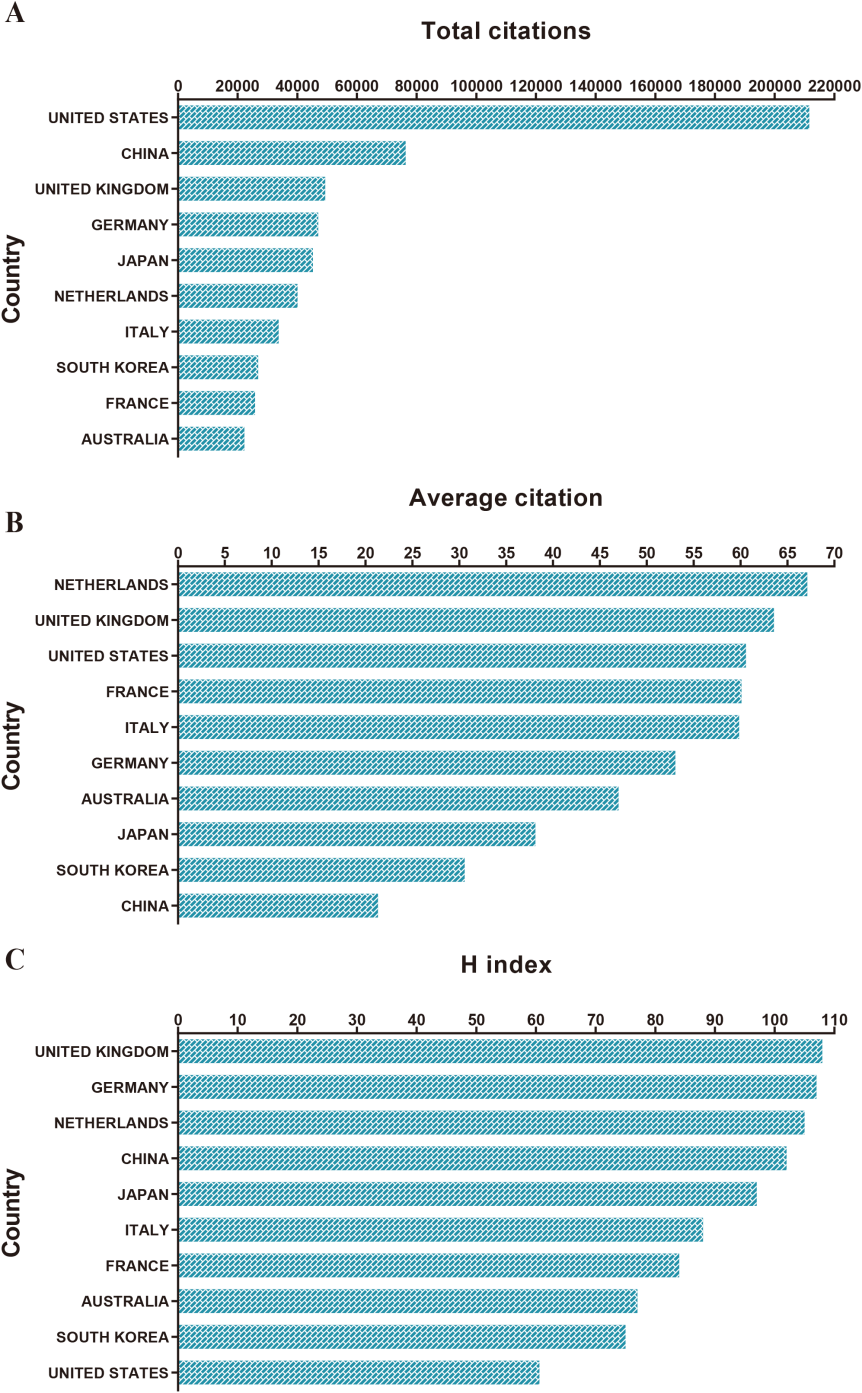
Total citation, Average Citation and H index levels of different countries/regions. **(A)** The top 10 countries/regions of total citations. **(B)** The top 10 countries/regions of the average citations per paper. **(C)** The top 10 countries/regions of the H-index.

According to the data illustrated in **Figure 3A**, publications from the United States have the highest cumulative citation frequency, exceeding 200,000 citations. China follows closely in second place, while the United Kingdom ranks third, with Germany and Japan in fourth and fifth positions, respectively. Additionally, papers from the Netherlands exhibit the highest average citation count. Notably, the United Kingdom, the United States, France, and Italy are ranked second, third, fourth, and fifth, respectively, in terms of average citation counts (**Figure 3B**). The statistical data shown in **Figure 3C** reveals that publications from the United Kingdom possess the highest H-index, followed in descending order by Germany, the Netherlands, China, and Japan. Although the Netherlands has the highest average citation frequency, it ranks third among countries for the highest H-index.

### 3.3 Analysis of the performance of countries and organizations

**Figure 4:**
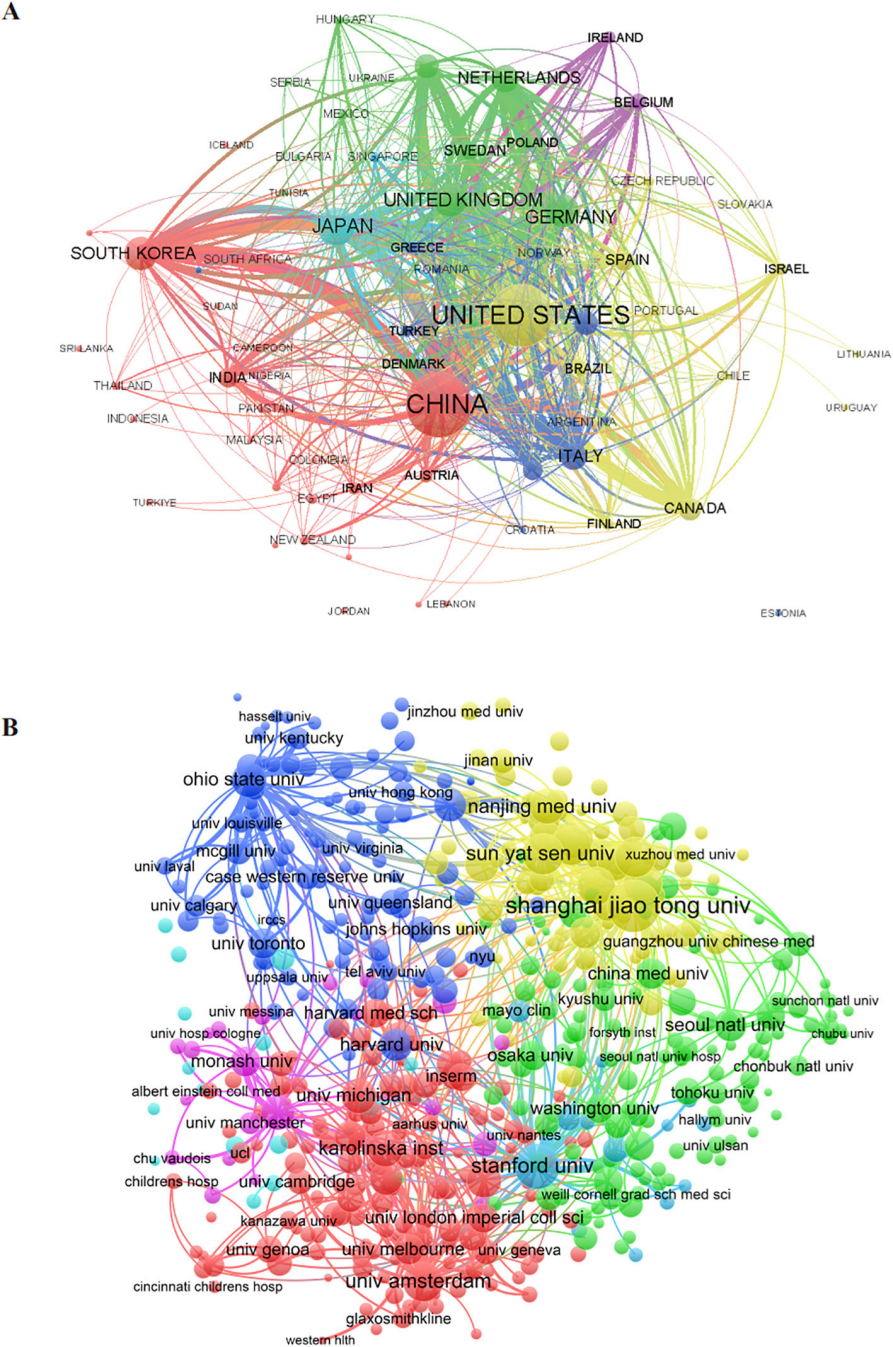
Globally leading countries and institutions. **(A)** Bibliometric network of production by country. **(B)** Bibliometric network of production by research institutions.

Open-access research on musculoskeletal (MSK) diseases involves contributions from 112 countries and regions. To examine collaboration within this domain, a country clustering map was created (**Figure 4A**), where each country or region is represented by a distinct sphere. The color of each sphere indicates the clustering relationships among research areas, with spheres grouped into clusters based on collaboration levels in those regions. The size of each sphere reflects the number of publications from that country. As illustrated in **Figure 4A**, red predominantly represents Asian countries, yellow signifies most American countries, and green denotes the majority of European countries, with these color distinctions primarily attributed to geographical factors. The United States and China exhibit the highest number of authors and the most substantial collaborative efforts. Specifically, China leads with 3,576 papers, accounting for 27.383% of the total, closely followed by the United States with 3,494 papers, representing 26.755% of the total. Japan ranks third with 1,187 contributions, accounting for 9.09% of the total.

The exploration of research and collaboration patterns among countries and regions in the context of “macrophage-MSK” offers a valuable resource for researchers and industry professionals. This information not only aids in steering collaborative initiatives but also helps identify key contributors within the field. Furthermore, it establishes a strategic basis for informed decision-making in research and development programs focused on MSK macrophages.

In the field of “Macrophages and Musculoskeletal Diseases,” international collaboration through specific forms such as joint laboratories and cross-border funding has played a crucial role in advancing research. This collaborative model not only facilitates scientific progress but also strengthens academic exchange and cooperation across different countries and regions.

Firstly, the establishment of joint laboratories provides researchers with a platform to share resources and technologies, enabling scientists from different countries to collaborate in the same experimental environment. This form of collaboration has demonstrated significant potential in the biomedical field. For example, the RA-MAP Alliance is a successful case, gathering more than 140 members from 21 academic and industrial organizations, aiming to achieve genomic medicine for rheumatoid arthritis (11). This cooperation not only enhances the quality and efficiency of research but also promotes the exchange of knowledge and technology transfer between different countries and regions, helping to better integrate global resources and drive scientific progress and innovation.

Secondly, in terms of cross-border project funding, such collaboration not only accelerates scientific research but also improves the quality and efficiency of research through resource sharing and knowledge exchange. Cross-border cooperation projects often attract more funding, which is particularly crucial for resource-limited research fields. For instance, a collaboration project in Ethiopia involved the orthopedics departments of the University of Utah and the University of California, Los Angeles, in partnership with Soddo Christian Hospital, demonstrating the importance of international cooperation in resource-scarce environments. This collaboration not only provided valuable experience to visiting residents and faculty but also brought significant benefits to local surgeons and trainees (12). Through such collaborations, researchers can validate their findings in different social and cultural contexts and assess which interventions are most effective (13). This not only helps overcome resource constraints but also fosters scientific advancement through multidisciplinary cooperation and cross-border funding.

Finally, international collaboration can also promote the development of scientific research by encouraging interdisciplinary cooperation. In the field of bone biology, interdisciplinary team science collaborations have already shown their importance, helping to drive innovation and progress in scientific research (14). In the field of bone immunology, international collaboration has contributed to the understanding of the complex interactions between bones and the immune system. Research has demonstrated that bone infections and inflammation lead to the infiltration of immune cells at the infection sites and regulate bone cell differentiation and function through the secretion of various cytokines and signaling mediators (15). This interdisciplinary cooperation provides a foundation for the development of new therapeutic approaches.

In conclusion, international cooperation has played a critical role in the development of the “Macrophages and Musculoskeletal Diseases” field. Through joint laboratories, cross-border funding, and interdisciplinary collaboration, international cooperation has not only advanced scientific research but also strengthened academic exchange and cooperation across countries and regions. This collaborative model offers new ideas and methods for future scientific research (16–18).

Over the last two decades, a total of 7,452 institutions have engaged in research concerning macrophages within the framework of MSK diseases. To visually depict the collaborative efforts among these research institutions, a collaboration clustering map was created (**Figure 4B**). These institutions are categorized into distinct clusters based on their cooperation levels. In this visualization, each sphere-text combination represents an institution, with the thickness of the connecting lines between spheres reflecting the degree of collaboration, and the size of each sphere indicating the number of publications from that institution. As outlined in **Table 1**, Shanghai Jiao Tong University has published the most papers (n = 292), representing 2.236% of the total, followed by the University of California system (n = 259, 1.983%) and the Ohio University system (n = 244, 1.868%).

**Table 1:** The top 10 institutions published literature related to macrophage and musculoskeletal system diseases from 2004 to 2023.

Rank	Institution	Article counts	Percentage	Country
1	SHANGHAI JIAO TONG UNIVERSITY	292	2.236	China
2	UNIVERSITY OF CALIFORNIA SYSTEM	259	1.983	United states
3	UNIVERSITY SYSTEM OF OHIO	244	1.868	United states
4	INSTITUT NATIONAL DE LA SANTE ET DE LA RECHERCHE MEDICALE INSERM	237	1.815	China
5	HARVARD UNIVERSITY	225	1.723	United states
6	CHINESE ACADEMY OF SCIENCES	215	1.646	China
7	US DEPARTMENT OF VETERANS AFFAIRS	207	1.585	United states
8	VETERANS HEALTH ADMINISTRATION VHA	204	1.562	United states
9	STANFORD UNIVERSITY	194	1.485	United states
10	UNIVERSITY OF LONDON	194	1.485	United kingdom

This extensive information not only provides insights into the dynamics of collaboration but also acts as a valuable resource for identifying opportunities for cooperation and establishing potential research partnerships. Our analysis emphasizes the potential influence of regional collaboration networks on local research initiatives and industry partnerships. These insights can offer significant guidance for industry professionals looking to engage with key research institutions, whether for joint ventures, clinical trials, or technology transfers.

### 3.4 Authors’ analysis

In the realm of open access, we analyzed author information and identified a total of 51,085 contributors in the “macrophage-MSK” domain. Notably, 919 authors exhibited significant productivity, having each published at least 10 papers. These findings highlight the key contributors in the “macrophage-MSK” field and present researchers with opportunities to tap into their expertise and insights. Understanding the substantial contributions of these authors can foster opportunities for collaboration and mentorship. The top 10 authors collectively published 906 papers, which represents approximately 6.935% of the total publications in this field. Wang Y is the most prolific author, with 151 papers, followed by Zhang Y with 125 papers, and Liu Y with 115 papers (**Table 2**).

**Table 2:** The top 10 authors with the most publications on macrophage and musculoskeletal system diseases from 2004 to 2023.

Rank	Highly Published Authors	Article counts	Percentage (%)
1	Wang Y	151	1.156
2	Zhang Y	125	0.957
3	Liu Y	115	0.88
4	Li J	107	0.819
5	Tak PP	95	0.727
6	Goodman SB	85	0.651
7	Li Y	84	0.643
8	Wang J	75	0.574
9	Wang L	69	0.528
10	Zhang L	68	0.521

**Figure 5:**
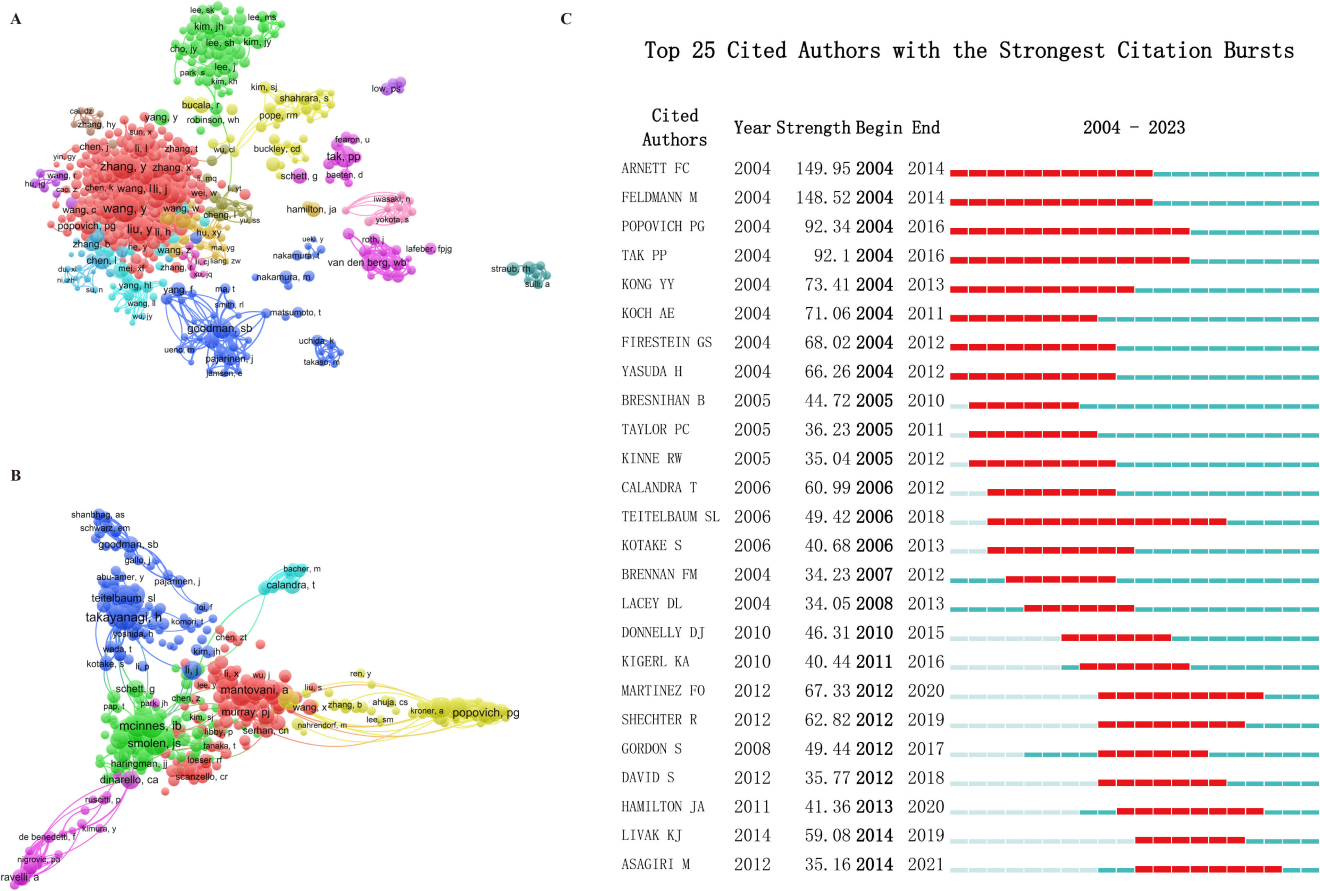
Globally leading author. **(A)** Network map of co-authorship by author. **(B)** Network map of co-cited by author. **(C)** Top 25 Cited Authors with the Strongest Citation Bursts.

To visualize the co-authorship network, VOSviewer software was employed, with **Figure 5A** depicting the strength of collaboration among different authors within the same field. Authors from the same country tend to collaborate more frequently and maintain stronger connections, whereas interactions between authors from different countries are generally limited. The generated visualization employs circles of varying sizes to indicate the number of documents authored by each individual, with different colors representing distinct clusters. The thickness of the connecting lines between the circles illustrates the strength of collaboration.

In the co-authorship analysis of the “Macrophages and Musculoskeletal Diseases” field, high-output authors have formed academic alliances and research centers, and this collaborative model has played a significant role in promoting scientific innovation. As scientific research becomes increasingly interdisciplinary, the boundaries between academia and industry have gradually blurred, with both sides developing outsourcing models to establish innovative collaborations and open funding opportunities (19). This cooperation extends beyond academia, including collaborations with the industry to optimize the process from discovery to innovation, requiring dynamic and multifaceted teamwork at the interface between universities and industry (20).

This cooperative model not only advances scientific research but also promotes the development of new technologies and methods. The International Society for Musculoskeletal Imaging (ISEMIR) facilitates global academic exchange through its annual meetings, discussing the application and research progress of imaging in rheumatology (21, 22). These conferences provide a platform for experts from around the world to share the latest research findings and technological advancements. Additionally, the RA-MAP Alliance serves as a successful case, bringing together over 140 members from 21 academic and industrial organizations, aiming to achieve genomic medicine for rheumatoid arthritis (11).

Furthermore, the “freshness” of a team, that is, the lack of prior collaboration among team members, has been shown to be closely related to the originality and interdisciplinary impact of research. Studies indicate that papers written by “fresher” teams tend to exhibit greater originality and multidisciplinary influence, and this effect is more pronounced in larger teams (23). This freshness is not only reflected in the new members joining the team but also in the novelty of their careers, which correlates with the originality and multidisciplinary nature of the papers produced.

In academia, collaborative research projects are also seen as an important way to unleash the potential of early-career scientists. Europe has launched several collaborative research projects specifically targeting early-career researchers, uniting a large number of them and generating a positive impact on the entire scientific community (24). This collaborative model also reflects the key role of scientific publications and presentations in establishing academic careers (25).

Finally, as research becomes more team-based and interdisciplinary, the recognition of authorship has become increasingly important. To improve transparency and reduce bias, some journals have started requiring explanations of how the first author position is determined among contributing authors (26). This practice not only helps to fairly acknowledge contributions but also promotes teamwork and innovation.

Co-citation analysis evaluates the relevance of items based on the frequency with which they are cited together. Using VOSviewer, we conducted an analysis of authors (**Figure 5B**). A significant metric known as “citation bursts” indicates how often authors are cited within a specific field during a designated timeframe. **Figure 5C** displays the top 25 most cited authors in “macrophage-MSK” research. Arnett FC leads substantially with a citation burst strength of 149.95, followed by Feldmann M, while Popovich PG ranks third with a burst strength of 92.34. Noteworthy authors such as Martinez FO, Hamilyon JA, and Asagiri M have also experienced a marked increase in their published papers in recent years, reflecting their dedicated contributions to the field.

These highly cited authors not only offer valuable insights into the “macrophage-MSK” field but also act as benchmarks for steering future research directions. This section presents a wealth of information for researchers and industry professionals, assisting them in navigating the intricate landscape of “macrophage-MSK” research. It helps in identifying key contributors, exploring collaborative opportunities, and staying updated on significant trends within the field.

### 3.5 Analysis of journals and their respective fields

**Figure 6:**
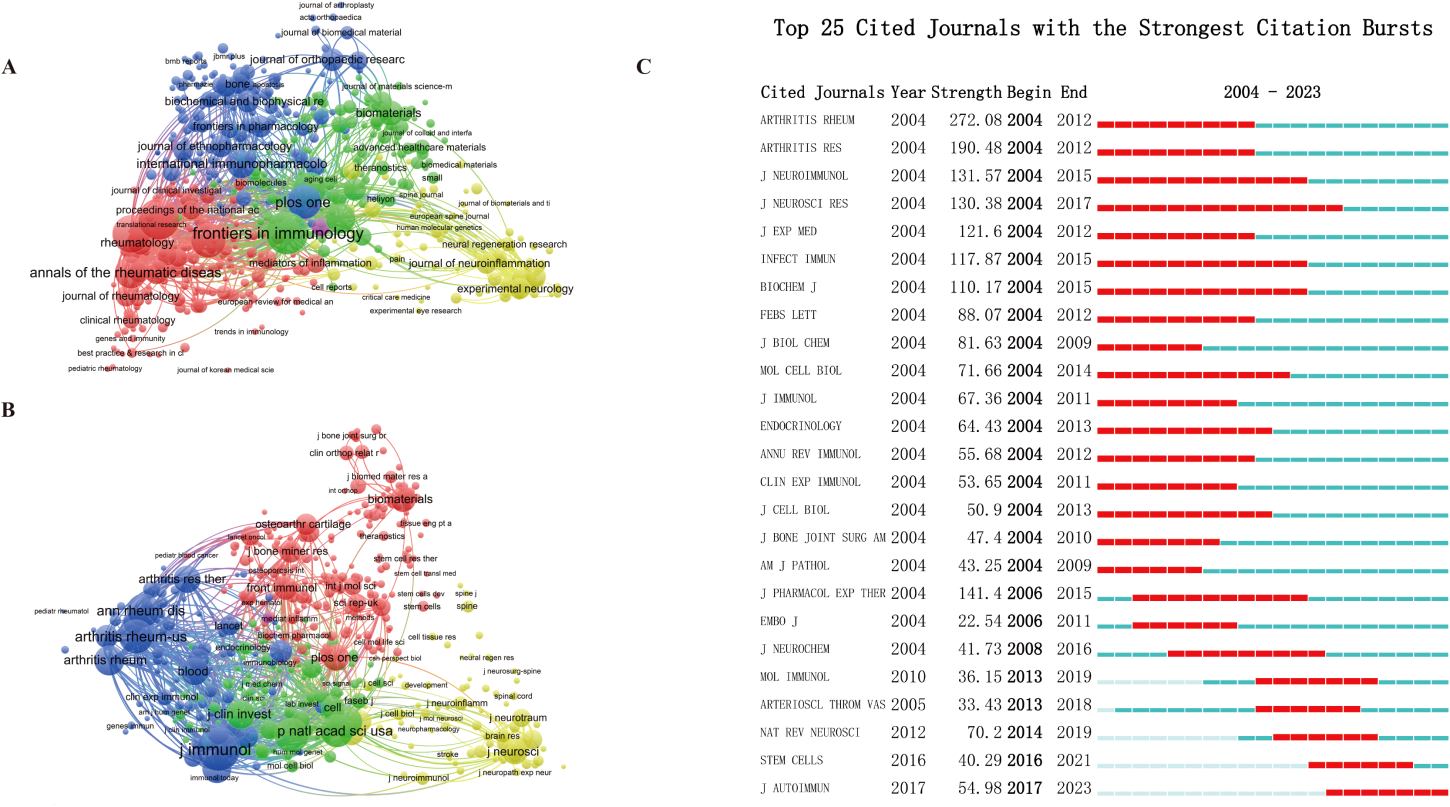
Globally leading journals. **(A)** Bibliometric network of production by journals. **(B)** Network map of co-cited journals. **(C)** Top 25 Cited Journals with the Strongest Citation Bursts.

To visualize the publication patterns of journal articles, we created a clustering map (**Figure 6A**). The distinctly colored areas represent specific clustering characteristics, with the sizes of the circles corresponding directly to the number of papers published by each journal. The top ten journals by article count in this field are listed in **Table 3**, with *Frontiers in Immunology* leading with the highest number of publications (n=343), followed closely by *Arthritis Research & Therapy* (n=332) and *Arthritis and Rheumatism* (n=273). Together, these top ten journals account for 17.142% of the overall publication total. A co-citation analysis of journal names was conducted using VOSviewer, which identifies the most frequently cited journals. As illustrated in **Figure 6B**, the total link strength of various journals is displayed, emphasizing their interconnections within the research domain.

**Table 3:** The top 10 most productive journals related to macrophage and musculoskeletal system diseases from 2004 to 2023.

Rank	Journal	Article counts	Percentage	Impact factor (IF, 2023)
1	FRONTIERS IN IMMUNOLOGY	343	2.626	5.7
2	ARTHRITIS RESEARCH THERAPY	332	2.542	4.4
3	ARTHRITIS AND RHEUMATISM	273	2.09	11.4
4	JOURNAL OF IMMUNOLOGY	233	1.784	3.6
5	PLOS ONE	222	1.7	2.6
6	INTERNATIONAL JOURNAL OF MOLECULAR SCIENCES	204	1.562	4.9
7	ANNALS OF THE RHEUMATIC DISEASES	191	1.462	20.3
8	SCIENTIFIC REPORTS	168	1.286	3.8
9	INTERNATIONAL IMMUNOPHARMACOLOGY	154	1.179	4.8
10	BIOMATERIALS	119	0.911	12.8

Additionally, we compiled a list of the top 25 most-cited journals pertinent to “macrophage-MSK” research (**Figure 6C**). This information is essential for researchers, as it aids in identifying key platforms for disseminating their findings and staying informed about the latest research trends.

In our investigation of macrophages associated with MSK conditions, we identified the top ten most representative fields (**Table 4**). The predominant field is immunology, which comprises 16.745% of the total, followed by rheumatology at 13.131% and pharmacology at 12.564%. This distribution suggests that the occurrence, progression, and treatment of MSK diseases are closely linked to macrophage-mediated immune responses. Macrophages are critical players in inflammatory processes, particularly in chronic inflammation and autoimmune diseases. Consequently, conducting in-depth research on the role of macrophage-mediated immunity in MSK diseases could unveil new approaches and strategies for their treatment (27)。

**Table 4:** The top 10 well-represented research areas.

Rank	Research Areas	Records	Percentage (%)
1	Immunology	2187	16.745
2	Rheumatology	1715	13.131
3	Pharmacology Pharmacy	1641	12.564
4	Cell Biology	1545	11.829
5	Biochemistry Molecular Biology	1408	10.78
6	Neurosciences Neurology	1123	8.598
7	Science Technology Other Topics	973	7.45
8	Research Experimental Medicine	950	7.274
9	Materials Science	924	7.074
10	Engineering	686	5.252

### 3.6 Co-Cited references analysis

**Figure 7:**
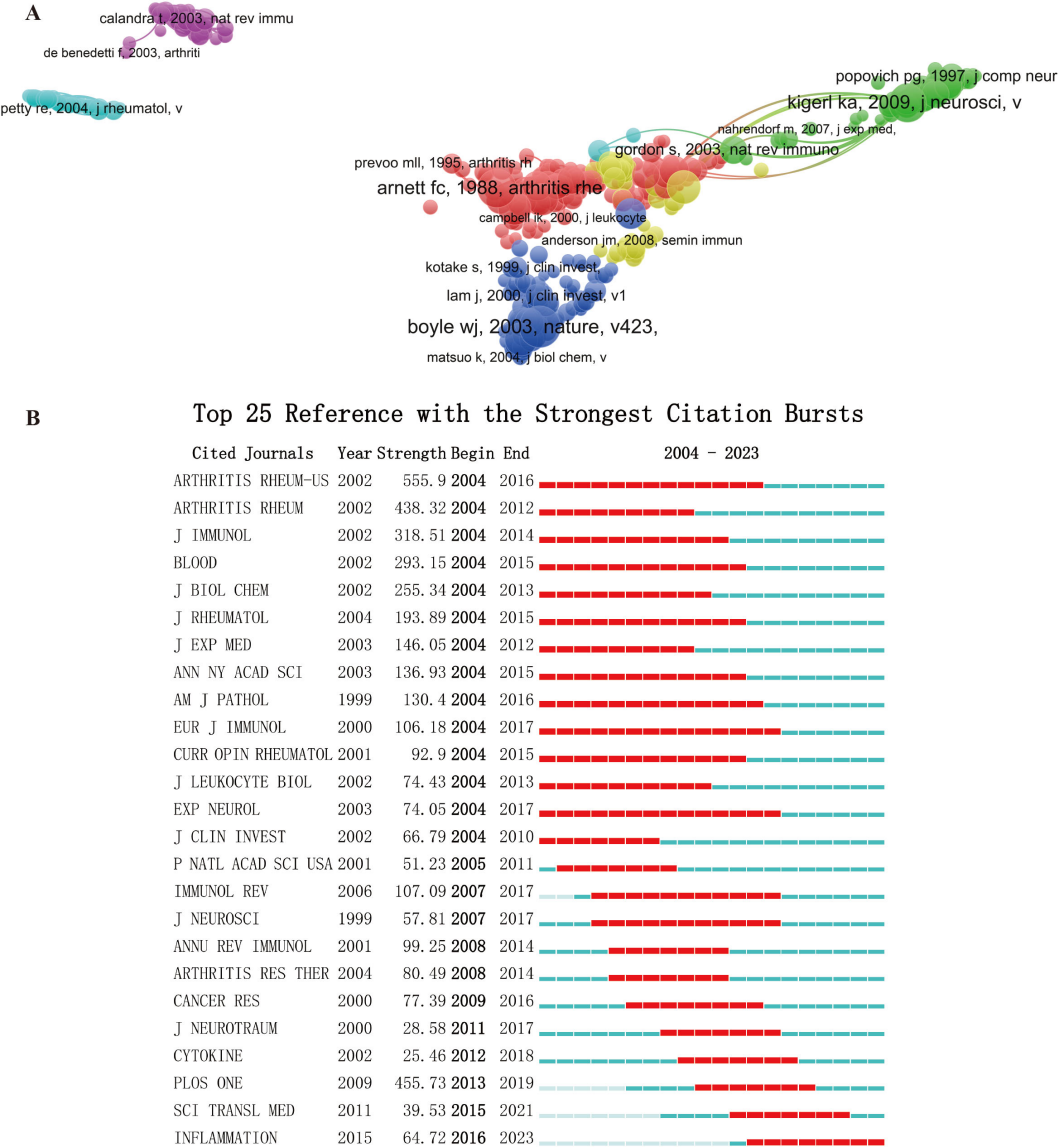
**(A)** Network map of co-citation analysis of references **(B)** Top 25 references cited most frequently in publications related to MKS-macrophages.

Using VOSviewer analysis (**Figure 7A**), we identified the most impactful publications in the field. Citation frequency serves as a valuable metric, reflecting the references that capture the interest of researchers over time. The detection of citation bursts by CiteSpace effectively highlights studies that have received significant attention. In our analysis, CiteSpace identified the top 25 journals with the strongest citation bursts, as presented in **Figure 7B**. Furthermore, the duration of these citation bursts is illustrated in **Figure 7B**, indicating the period during which these citations sustained high levels of visibility.

The most frequently cited reference is the 1987 revision of the rheumatoid arthritis classification criteria by the American College of Rheumatology, which was established based on a computer analysis of 262 rheumatoid arthritis patients and 262 non-rheumatoid rheumatoid disease patients (28). This standard has had a profound impact: it standardized diagnostic methods in the early stages, improved diagnostic accuracy, reduced misdiagnoses and missed diagnoses; the introduction of the new criteria provided a unified framework for clinical research on RA, making results from different studies more comparable and enhancing the reliability of RA research; with more accurate classification, doctors could diagnose early and take timely corresponding treatment measures, aiding in personalized treatment plans and improving treatment outcomes; the revision of these criteria provided a common framework for rheumatology research and clinical practice worldwide, facilitating consensus among researchers and physicians in different countries and regions, promoting international collaboration and information sharing, and advancing global RA research; by offering a standardized diagnostic criterion, the report also provided strong support for the formulation of medical policies and epidemiological studies of rheumatic diseases. Overall, this report, by proposing more stringent and accurate diagnostic criteria, not only improved the clinical diagnostic level of rheumatoid arthritis but also fostered global research collaboration, promoting the development of the RA field.

Next, a highly cited paper published in 2003 is also noteworthy. This study focused on osteoclast function, revealing that osteoclasts, as specialized cells derived from the monocyte/macrophage hematopoietic lineage, adhere to the bone matrix and secrete acids and enzymes to degrade bone tissue. The study identified the core signaling pathway—RANK pathway—essential for osteoclast development, activation, and bone resorption, providing a molecular foundation for understanding the mechanisms behind bone loss diseases such as osteoporosis. This discovery not only advanced the understanding of bone resorption mechanisms but also opened new directions for orthopedic clinical treatments, especially in drug development for diseases like osteoporosis. By targeting osteoclasts or the RANK pathway, researchers can effectively treat diseases related to bone resorption (29).

Furthermore, a 2009 paper on the role of macrophages in central nervous system injury, particularly in spinal cord injury, was also frequently cited. The paper revealed the different roles of M1 and M2 macrophages in injury repair after spinal cord injury. M1 macrophages are neurotoxic, whereas M2 macrophages promote regeneration and aid in repair. The study showed that the imbalance between M1/M2 macrophages affects the repair process of the central nervous system. The key finding of this study was that by polarizing macrophages towards the M2 type, it could promote nerve repair and limit the inflammation caused by M1 macrophages. This result provides new therapeutic strategies for central nervous system injuries, especially with significant clinical application prospects (30).

### 3.7 Analysis of keywords and hotspots

**Figure 8:**
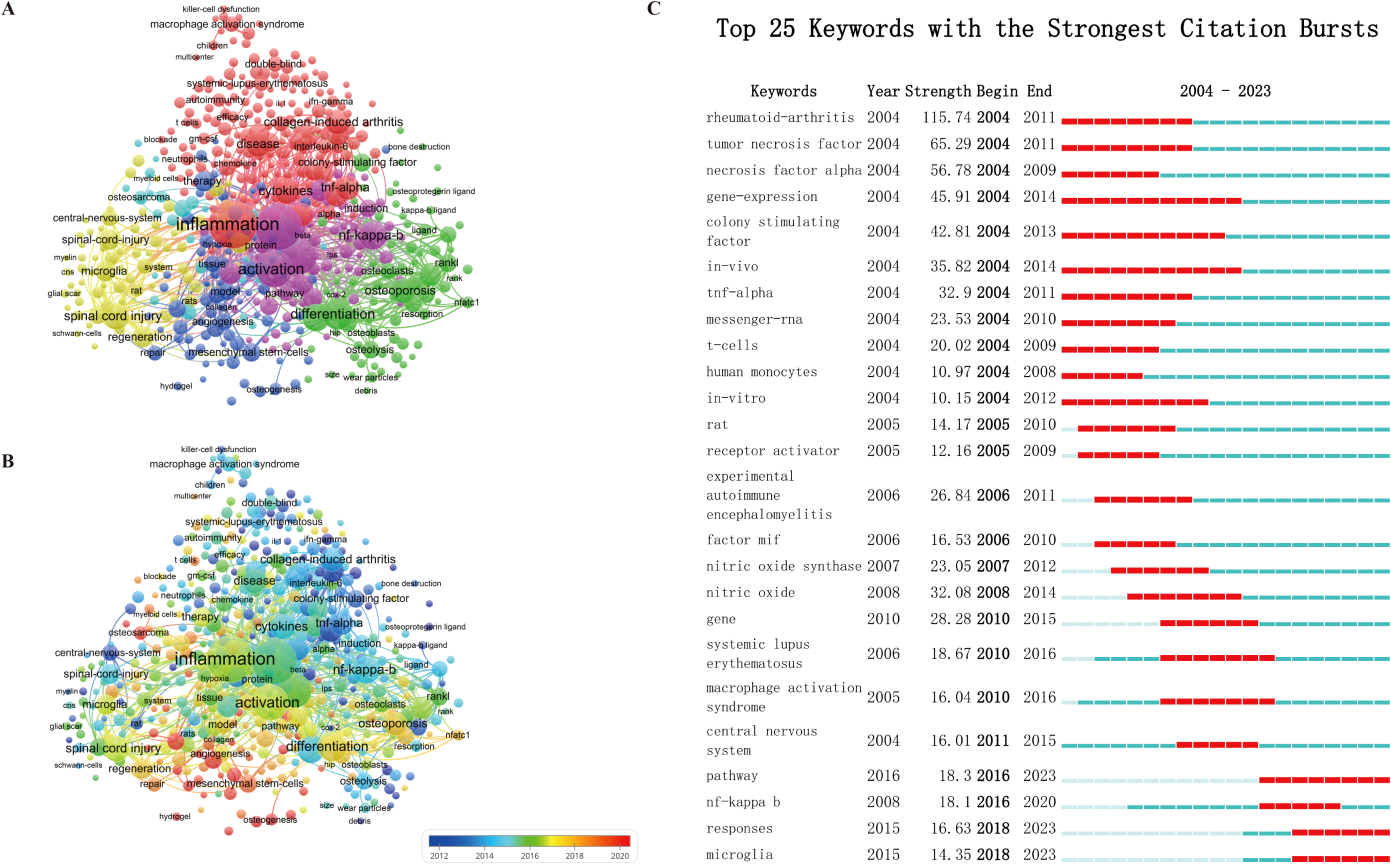
**(A)** Keyword mapping in MSK-macrophage-related research; the frequency is represented by the point size. **(B)** Keywords are distributed according to the average frequency; red keywords appear later than blue keywords. **(C)** Top 25 Keywords with the Strongest Citation Bursts.

In bibliometrics, keyword co-occurrence analysis is a widely employed method for identifying trending research topics and areas, serving a crucial function in tracking the progress of scientific inquiry. In this analysis, keywords are defined as terms that appear multiple times in the titles or abstracts of all selected publications, which are subsequently examined using VOSviewer. This approach yields five clusters, each representing one of the primary research directions within the field, as depicted in **Figure 8A**.

In **Figure 8B**, VOSviewer assigns colors to keywords according to their average appearance time in the published literature. Specifically, blue indicates that the keywords were introduced relatively early, while orange-red denotes more recent occurrences. The average publication years for these keywords range from 2012 to 2020. The primary keywords emerging from the average publication years of 2018 to 2020 include “repair” (150), “nanoparticles” (129), and “delivery” (128).

Using CiteSpace’s burst detection analysis, we can identify which keywords have gained significant attention over a certain period and further hypothesize why these topics have become research hotspots. This burst phenomenon reflects the academic community’s strong interest in certain issues and the importance of these topics during a specific timeframe. **Figure 8C** displays the top 25 keywords with the highest burst intensity.

First, “Rheumatoid arthritis” stands out as the keyword with the highest burst intensity (intensity = 115.74), indicating its recent emergence as a focal point in macrophage-related MSK disease research. Rheumatoid arthritis (RA) is a chronic inflammatory disease closely linked to immune system dysfunction, and macrophages play a central role in immune responses (31, 32). As research deepens into immune regulation and cytokine mechanisms, more scholars are focusing on the role of macrophages in RA, particularly in inflammation and joint destruction processes (33, 34). Research in this area not only focuses on the disease pathogenesis but is also shifting toward the exploration of novel therapeutic strategies, such as targeted immunotherapy and cell therapies (35–37). Thus, rheumatoid arthritis has become the leader in the burst keyword phenomenon.

Next, “tumor necrosis factor” (intensity = 65.29) and “necrosis factor alpha” (intensity = 56.78) follow closely, reflecting the field’s attention to the role of inflammatory factors. TNF-α is an important inflammatory factor that is widely involved in the development of various immune-related diseases (38). Its role in MSK diseases like RA, especially in regulating macrophage activation and inflammation, has triggered numerous studies. With the clinical success of anti-TNF therapies (e.g., biologics), research on TNF-α has experienced rapid growth (39). Researchers are not only focusing on its pathological role in diseases but also exploring how to target and inhibit its activity to slow or halt disease progression, making “tumor necrosis factor” a research hotspot (40).

“Gene expression” and “*in vivo*” are two keywords that top the list for burst duration, each lasting for ten years, indicating they have remained central topics in research during this period. Gene expression is a key aspect of cellular biological processes that directly relate to macrophage regulation in MSK diseases (41). Studies have shown that macrophage function and polarization play a crucial role in MSK diseases, and the regulation of these functions depends on gene expression and transcriptional regulation (42–44). Therefore, understanding how gene expression changes in macrophage function, especially in various MSK diseases, has become a primary research focus in this field.

Additionally, keywords like “Pathway,” “Microglia,” and “Responses” have also shown burst citation phenomena recently, suggesting they may become important research directions in the future. Macrophages and microglia, two major cell populations in the immune system, are receiving increasing attention for their role mechanisms in various diseases (45–47). Specifically, in MSK disease immune responses, macrophages and microglia regulate immune responses, inflammation, and tissue repair through different signaling pathways. By exploring how these cells interact post-injury and their roles in disease progression, researchers are providing potential directions for new therapeutic approaches (48–50).

The appearance of these burst keywords is not coincidental but is closely related to the deepening understanding of specific issues in the academic community and the continuous advancement of technological methods. First, with the ongoing development of immune system and inflammation mechanism understanding, scholars have gradually recognized the essential roles of macrophages and other immune cells in MSK diseases. Second, advancements in biomedical technologies, particularly genomics, molecular biology, and high-throughput screening, have enabled researchers to more accurately reveal the specific mechanisms of macrophages in MSK diseases, driving the rise of related research.

**Figure 9:**
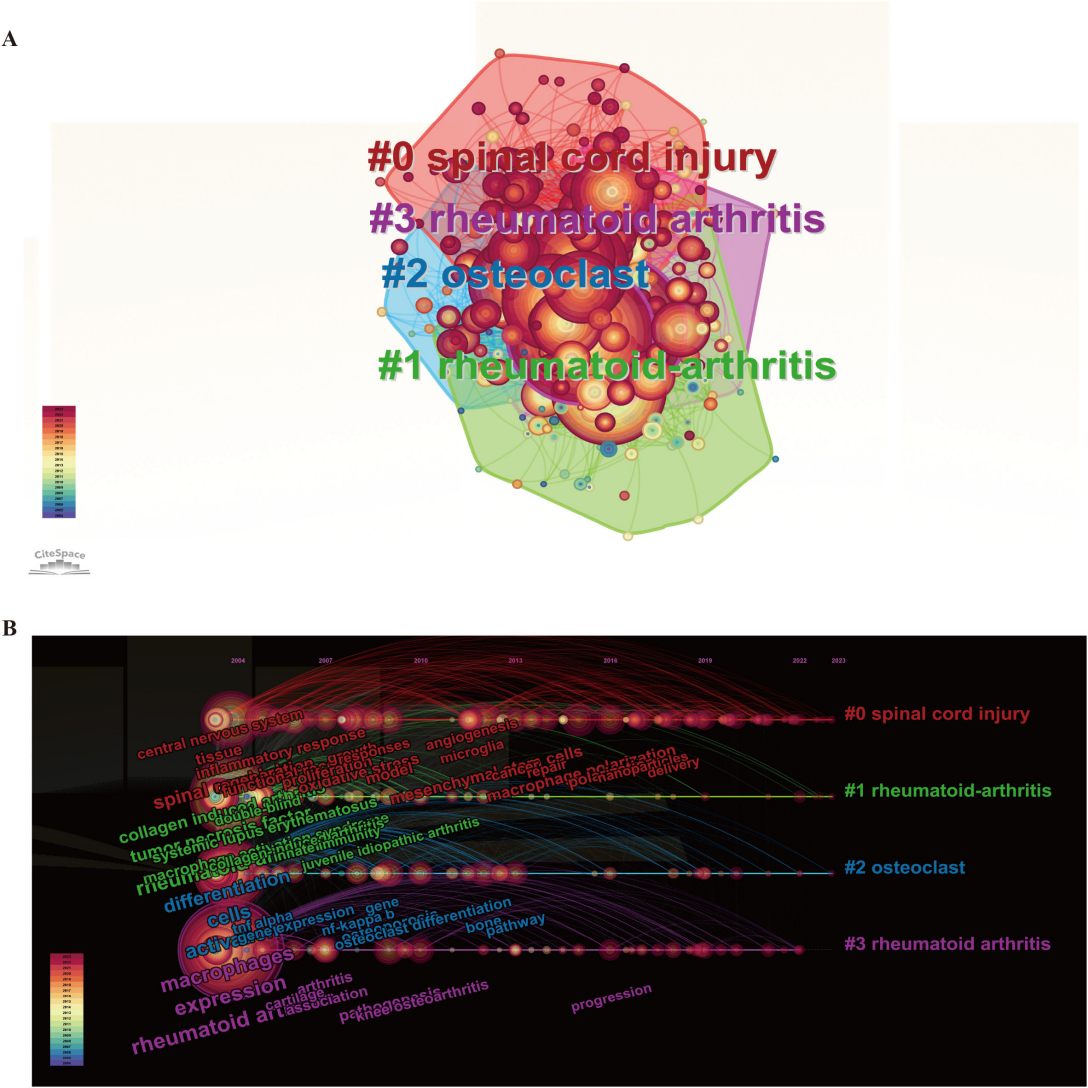
Bibliometric analysis of keywords and orientation in the future: **(A)** Clustering analysis of the keyword network based on CiteSpace. **(B)** Timeline diagram of keywords with corresponding changes of cluster.

We also created a network graph to visually illustrate keyword clusters (**Figure 9A**). The analysis revealed that “spinal cord injury” (Cluster 0), “rheumatoid arthritis” (Cluster 1), “osteoclast” (Cluster 2), and “rheumatoid arthritis” (Cluster 3) have emerged as significant research hotspots since 2004. The top ten most representative research fields are detailed in **Table 4**, with “Immunology” taking the lead, followed by “Rheumatology” and “Pharmacology and Pharmacy.” By performing a temporal cluster analysis of keywords, we can establish a foundation for research topics in this field. To effectively pinpoint turning points and emerging trends within the discipline, the keyword co-occurrence map can be organized chronologically, illustrating the distribution of research hotspots across different periods.

As depicted in **Figure 9B**, the “macrophage-MSK” field has evolved globally over the past 20 years, categorized into seven distinct phases. Phase 1 (2004-2007): The research primarily centered on diseases, target organs, and the intrinsic functions of macrophages. Keywords such as “expression,” “activation,” and “differentiation” were prominent during this period, reflecting the close relationship between the progression of musculoskeletal (MSK) diseases and the expression of specific proteins by macrophages, activation of signaling pathways, and their differentiation processes. Phase 2: The focus shifted to keywords like “mechanisms,” “regeneration,” and “pathogenesis,” indicating an increased interest in understanding the roles of macrophages in the context of MSK diseases. This period saw growing public attention towards the pathogenesis of macrophages and their influence on regeneration. Phases 3, 4, and 5 (2011-2019): The research landscape featured keywords such as “mesenchymal stem cells,” “oxidative stress,” “repair,” and “macrophage polarization.” This highlights a marked emphasis on functional studies of macrophages and their impact on regenerative engineering approaches within MSK diseases. Phases 6 and 7 (2020-2023): The keywords that emerged most frequently included “nanoparticles,” “delivery,” “bone regeneration,” and “extracellular vesicles.” The expanding breadth of research suggests a growing focus on regenerative medicine, targeted delivery strategies, and the exploration of various biomaterials, particularly nanomaterials. This indicates a convergence of scholars from diverse backgrounds, with a shift in research from mechanistic exploration towards translational medicine.

## 4 Discussion

In an age characterized by information overload, staying updated on industry advancements and the latest research findings has become increasingly difficult. To effectively communicate the current state of research worldwide, particularly in the area of “macrophage-based musculoskeletal (MSK) research” from 2004 to 2023, we employed bibliometric analysis. This analytical approach is instrumental in clarifying and organizing the research framework within a specific field while also predicting future trends. Additionally, it plays a crucial role in evaluating the potential impact of upcoming investigations related to macrophages in MSK studies.By systematically analyzing publication patterns, citation metrics, and collaboration networks, bibliometric analysis offers insights into key contributors, emerging topics, and opportunities for further exploration. This not only enhances our understanding of the current landscape but also guides future research directions and strategies in the rapidly evolving field of macrophage research within musculoskeletal diseases.

### 4.1 General information

A bibliometric analysis was performed on 13,687 publications associated with “macrophage-based MSK research” spanning from 2004 to 2023, retrieved from the WoSCC database. This investigation uncovered several noteworthy and important trends. Over the past two decades, global research production in this domain has shown a consistent upward trajectory, with projections indicating continued growth in interest through 2024. The foundational studies conducted in the early stages of this field have established a solid basis for subsequent advancements, and the rising volume of scholarly work points toward a promising future for this area of research.

A keyword analysis revealed that studies focused on macrophages in rheumatoid arthritis represent the largest share within the “macrophage-based MSK research” field. This emphasizes the pivotal role of macrophages in the onset, progression, and treatment of rheumatoid arthritis. Furthermore, the contributions of various institutions and funding sources have played a significant role in shaping research output, forming a crucial foundation for the field’s growth. The majority of participating institutions are universities, indicating that current efforts are primarily concentrated on basic research, while translational medicine remains underexplored. Looking ahead, it is expected that more hospitals will engage in clinical research, thereby contributing to the resolution of this global health challenge.

As indicated in **Table 5**, the “National Natural Science Foundation of China (NSFC)” has supported the largest number of publications, underscoring its essential role in advancing scientific research in China, particularly in the realms of basic and applied studies. Analyzing NSFC-funded projects reveals its significant contribution to the development of research teams, the cultivation of young scholars, and the encouragement of exploratory studies (51, 52). This support has not only enhanced China’s prominence on the global research platform but also laid the scientific groundwork and provided technical backing for addressing critical health challenges both domestically and internationally. The NSFC serves not only as a financial pillar for research endeavors but also as a driving force behind China’s scientific and technological advancement.

**Table 5:** The top 10 funds related to macrophage and musculoskeletal system diseases from 2004 to 2023.

Rank	Journal	Article counts	Percentage
1	NATIONAL NATURAL SCIENCE FOUNDATION OF CHINA NSFC	2078	15.91
2	UNITED STATES DEPARTMENT OF HEALTH HUMAN SERVICES	1768	13.536
3	NATIONAL INSTITUTES OF HEALTH NIH USA	1757	13.452
4	MINISTRY OF EDUCATION CULTURE SPORTS SCIENCE AND TECHNOLOGY JAPAN MEXT	490	3.752
5	JAPAN SOCIETY FOR THE PROMOTION OF SCIENCE	436	3.338
6	GRANTS IN AID FOR SCIENTIFIC RESEARCH KAKENHI	393	3.009
7	NIH NATIONAL INSTITUTE OF ARTHRITIS MUSCULOSKELETAL SKIN DISEASES NIAMS	340	2.603
8	EUROPEAN UNION EU	261	1.998
7	GERMAN RESEARCH FOUNDATION DFG	259	1.983
10	NATIONAL RESEARCH FOUNDATION OF KOREA	248	1.899

In terms of authorship, Wang Y leads with 151 publications, while Tak PP stands out for having the highest average citation count among the top ten, with a primary focus on rheumatoid arthritis (RA) research (53, 54). RA, a complex autoimmune condition, has witnessed substantial advancements in both research and treatment over recent years. A bibliometric analysis of RA-related literature demonstrates Tak PP’s considerable influence on both the academic understanding and clinical management of the disease (55). This underscores the growing focus on macrophage-related research within MSK disorders, particularly RA. As one of the most prominent MSK diseases, RA has garnered significant attention from researchers investigating the role of macrophages. Studies have shown that the accumulation of macrophages within the synovial cavity of RA patients is closely associated with the disease’s inflammatory response. Notably, the proportion of pro-inflammatory M1 macrophages is markedly higher than that of anti-inflammatory M2 macrophages, which results in elevated secretion of inflammatory cytokines and an intensification of inflammation. This underscores the critical role of macrophages, particularly their polarization, in RA pathology and suggests that targeting these cells may offer a promising therapeutic strategy (56).

Academic journals are essential platforms for the dissemination of scientific research, with their quality and reputation being key factors in the effective spread of scientific knowledge. Among the leading journals, three of the top ten have impact factors (IF) exceeding 10.000, highlighting their substantial influence within the academic community. In the field of immunology, researchers often prioritize “MSK-based macrophage research” over topics typically covered by molecular biology or pharmacology journals. This trend likely reflects the current research priorities, particularly the focus on understanding immune responses and the mechanisms driving related diseases. Additionally, the journal *Biomaterials*, with an IF of 12.8, signals the high expectations within the academic community for advancements in molecular research in this area.

The journals most frequently cited within a specific timeframe reflect the foundational knowledge and contextual framework of a field, often marking key milestones in the evolution of certain topics. As demonstrated in **Figure 7B**, *Arthritis & Rheumatology* is the most-cited journal, underscoring that clinical research and guidelines published in high-impact journals are more likely to be referenced. This highlights the critical role these journals play in advancing both medical knowledge dissemination and clinical practice. While some journals may have high publication volumes, their citation counts do not always align with this output. In recent years, despite the growing number of published papers, the academic community continues to prioritize high-quality research as its central focus.

### 4.2 Hotspots and frontiers

In scientometrics, keyword co-occurrence can reveal the key focus areas within an academic field, while timeline charts illustrate the evolution of emerging trends. Over the past two decades, the increasing number of keywords signals that macrophage research has become a prominent topic in the study of MSK diseases. Keyword clustering shows that researchers are not only concentrating on the primary origins of macrophages (such as embryonic or bone marrow sources) but are also investigating their differentiation processes in response to homeostatic cues, disease-related signals, and temporal factors. These areas are crucial for understanding the role of macrophages in either maintaining physiological homeostasis or driving disease progression (57). In Asian countries, as research on MSK diseases advances, the role of macrophages in these conditions has garnered growing interest (58). Consequently, macrophage research has become a critical area, not only in fundamental biology but also in clinical investigations and the development of therapeutic interventions.

**Figure 10:**
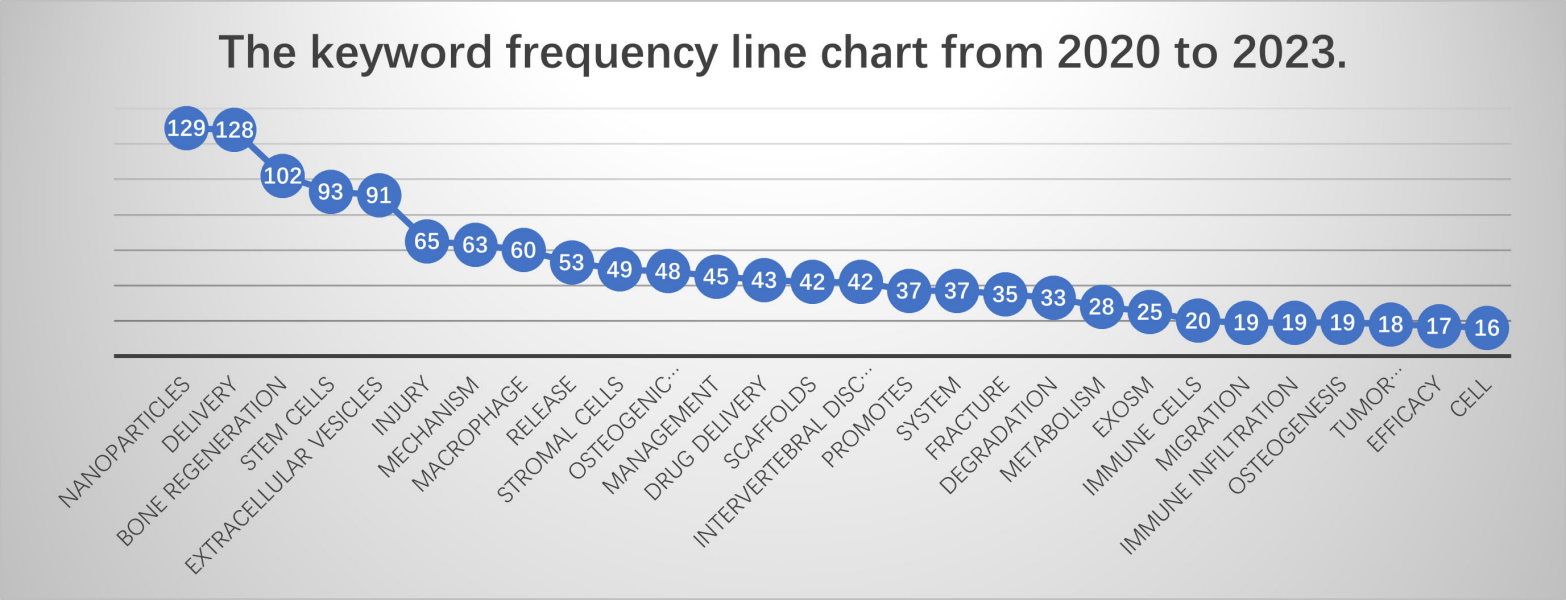
The keyword frequency line chart from 2020 to 2023.

As academic interest in this field continues to expand, the cognitive perspectives are broadening, with certain key terms maintaining their influence in current research. These keywords highlight the academic community’s focus on “MSK-based macrophage research.” The keywords from the past four years predominantly reflect the latest research objectives in this area. Drawing from the findings of this bibliometric analysis, we analyzed the most frequently occurring and research-relevant keywords over the past four years to identify the core areas of investigation. The frequency ranking of keywords in 4 years is shown in **Figure 10**.

#### 4.2.1 Nanoparticles

The involvement of nanoparticles in “MSK-based macrophage research” has been extensively documented in the literature. In recent years, the use of nanomaterials in medicine and biology has surged, particularly in studies of the immune system. As key players in the innate immune response, macrophages are crucial for defending the body against foreign substances (59). The interaction between nanoparticles and macrophages not only influences macrophage functionality but can also modify their responses to pathogens and immune regulation capabilities (60). For instance, studies on metal oxide nanoparticles have demonstrated their ability to modulate immune responses by altering macrophage receptor expression and signaling molecule secretion (59). Additionally, research on carbon dot nanoparticles has shown their high uptake efficiency by macrophages, coupled with low toxicity, positioning them as promising candidates for drug delivery and bioimaging (61). As investigations into nanoparticle-macrophage interaction mechanisms deepen, researchers are increasingly recognizing the therapeutic potential of these nanomaterials in disease treatment and prevention. For example, surface-modified nanoparticles can be designed to specifically target diseased cells, thereby improving therapeutic efficacy.

Nanoparticles have found extensive application in research related to MSK-based macrophages. A deeper understanding of the pathophysiology underlying chronic inflammatory diseases, such as rheumatoid arthritis and osteoarthritis, has revealed that the accumulation of diseased tissues and the overexpression of certain molecules, including macrophages, can be harnessed to enhance the delivery of nanomedicines. Nanodrugs can either passively accumulate in chronic inflammatory tissues due to enhanced permeability and retention effects or actively bind to receptors overexpressed on cells within these tissues through ligand surface conjugation, leading to increased efficacy and reduced systemic side effects (62, 63). In the context of immunotherapy for osteosarcoma, nanoparticles serve as effective drug delivery systems that specifically target tumor cells and potentially enhance anti-tumor immune responses by modulating macrophage function. Research has demonstrated that macrophages exhibit a dual role within the tumor microenvironment, promoting tumor growth while simultaneously inhibiting its progression through the release of cytokines and chemokines. For instance, Staphylococcus aureus has been shown to amplify macrophage inflammatory responses, counteracting the immunosuppression associated with osteosarcoma (64). This observation indicates that the design of nanoparticles could be optimized to enhance interactions with macrophages, ultimately improving therapeutic outcomes in osteosarcoma treatment.In studies of intervertebral disc (IVD) degeneration, the interplay between nanoparticles and macrophages has garnered considerable attention. IVD degeneration is often linked with an inflammatory response, in which macrophages play a critical role. Research suggests that the polarization state of macrophages (M1 or M2) significantly influences inflammation and regeneration within the disc. In the pathological context of IVD degeneration, an increase in M1 macrophages results in the secretion of pro-inflammatory cytokines, thereby exacerbating tissue damage (65, 66). To tackle this challenge, researchers have developed various nanoparticle systems aimed at achieving targeted drug delivery and regulating macrophage activity. For instance, polyamide-based nanoparticles have been utilized to deliver mRNA into human joint and disc cells, demonstrating high transfection efficiency in most cell types but showing limited expression in M1 macrophages (67). Moreover, bioinspired structured nanoparticles capable of adsorbing inflammatory factors and regulating autophagy have been developed to promote the transition of M1 macrophages to the M2 phenotype, thereby alleviating inflammation in the disc (68). Additionally, nanoparticles are increasingly recognized as an emerging therapeutic strategy in osteoporosis research. These nanoparticles specifically target macrophages to enhance drug bioavailability and pharmacokinetics (69). For example, calcium-polyCpG metal polymer DNA nanoparticles (Ca-polyCpG MDNs) have been created to reconstruct the osteoporotic microenvironment, inhibiting bone resorption and facilitating bone repair (70). These nanoparticles counteract the acidic conditions associated with bone resorption by providing calcium supplementation and promoting bone remineralization, ultimately improving treatment outcomes for osteoporosis. Furthermore, magnesium oxide nanoparticles (MgO NPs) have demonstrated promising immunomodulatory effects, inhibiting both osteoporosis and inflammation triggered by titanium particles (71). These nanoparticles regulate macrophage polarization, suppress bone resorption, and influence the bone remodeling process.

In conclusion, nanoparticles are receiving increasing attention in the realm of “MSK-based macrophage research.” This trend underscores the widespread application of nanomaterials in medical and biological investigations, especially regarding their effects on the immune system. Macrophages, as pivotal cells in the innate immune response, play a crucial role in the pathogenesis of various diseases (72). Research indicates that macrophages not only engage in immune responses during the initial phases of disease but also facilitate repair and regeneration in later stages (69, 72). Thus, a comprehensive understanding of how nanoparticles influence macrophage functionality is essential for the development of novel therapeutic strategies and the enhancement of existing treatments (59, 73). Furthermore, research focused on nanoparticle interactions with macrophages continues to evolve, with the distinctive properties of nanoparticles facilitating more efficient drug delivery and targeted therapies (69). This progress opens up new avenues for treating MSK diseases, particularly by leveraging the biological characteristics of macrophages to enhance drug bioavailability and therapeutic effectiveness.

#### 4.2.2 Bone regeneration

Bone regeneration is a multifaceted biological process that entails interactions among various cell types, with macrophages and mesenchymal stem cells (MSCs) playing pivotal roles. Macrophages serve a significant regulatory function in bone regeneration, influencing cellular metabolism and tissue repair through the secretion of cytokines and other bioactive substances (74). The polarization state of macrophages, such as M1 and M2 types, directly affects the bone healing process. M1 macrophages are typically linked to inflammatory responses, while M2 macrophages are recognized for their anti-inflammatory properties and their involvement in facilitating tissue remodeling (75). The presence and functionality of MSCs are also crucial in the bone regeneration process. MSCs possess the capability to differentiate into osteoblasts, thereby aiding in bone formation. Concurrently, macrophages regulate the transition of undifferentiated mesenchymal cells into osteoblasts by releasing specific proteins (76). Additionally, the origin of macrophages plays an important role; they can be derived from fetal red marrow progenitors or adult hematopoietic precursors, with the functions of these macrophages in bone healing potentially varying based on their source.

Research demonstrates that the phenotypic alterations of macrophages are intricately linked to the temporal dynamics of bone regeneration. In inflammatory bone resorption diseases, the pro-inflammatory activation of macrophages is associated with bone loss, whereas the activation of resolving macrophages following the removal of stimuli is closely tied to the restoration of bone levels (75). By modulating macrophage functions, it is feasible to effectively enhance bone regeneration and repair, presenting novel strategies for the treatment of MSK-related diseases (74).

#### 4.2.3 Stem cells

Skeletal muscle regeneration depends on the activation and proliferation of muscle stem cells, known as satellite cells, processes that are modulated by macrophages. Macrophages are integral to the repair mechanisms following muscle injury, as they influence satellite cell behavior through the release of cytokines and other signaling molecules. Research indicates that specific subpopulations of macrophages “reside” at the site of injury, providing a temporary supportive environment for satellite cells, which is vital for muscle regeneration (77). In the realm of stem cell therapy, mesenchymal stem cells derived from embryonic stem cells (ESC-MSCs) have been shown to effectively mitigate skeletal muscle damage resulting from acute compartment syndrome. ESC-MSCs facilitate muscle regeneration and functional recovery by modulating the polarization state of macrophages and attenuating inflammatory responses (78). Furthermore, extracellular vesicles derived from mesenchymal stem cells demonstrate promise in regulating macrophage phenotypes, representing a novel acellular approach for treating skeletal muscle diseases. These extracellular vesicles can impact macrophage function, modifying inflammatory processes to promote muscle repair and regeneration (79).

In summary, the interactions between stem cells and macrophages play a critical role in the treatment of skeletal muscle diseases. Future research is likely to shed light on these mechanisms, potentially offering new avenues for clinical applications.

#### 4.2.4 Extracellular vesicles

Extracellular vesicles (EVs) are recognized as a crucial medium for intercellular communication, particularly within the context of “MSK-based macrophage research,” underscoring their potential biological significance. Macrophages are integral not only to immune responses but also to tissue repair and regeneration processes. Recent investigations have shown that EVs released by macrophages can transport various bioactive molecules, including cytokines, miRNAs, and proteins, which are vital in the onset and progression of musculoskeletal diseases. In inflammatory conditions such as arthritis, EVs derived from macrophages are deemed essential factors in modulating inflammatory responses. They can affect the behavior of neighboring cells by conveying pro-inflammatory or anti-inflammatory signals, thereby assuming a dual role in the disease process (80).

In the context of musculoskeletal diseases, EVs can influence disease progression by modulating the polarization states of macrophages. For instance, EVs released from damaged tissues may promote the activation of M1 macrophages, thereby exacerbating inflammatory responses, while EVs originating from M2 macrophages could enhance tissue repair and regeneration. This intricate interplay of interactions positions EVs as potential therapeutic targets that can improve treatment outcomes for musculoskeletal diseases through the regulation of macrophage function. Moreover, EVs may also act as potential biomarkers for monitoring disease progression and treatment responses (81). Thus, investigating the role of EVs within the “MSK-macrophage” framework can offer significant theoretical insights and practical guidance for developing novel therapeutic strategies.

#### 4.2.5 Delivery

In recent years, the keyword “Delivery” has gained increasing attention in the “Macrophages and Musculoskeletal Diseases” field, reflecting the growing demand for precise drug delivery in this area. Macrophages play crucial roles in tissue homeostasis and the innate immune system, exhibiting abnormal polarization in diseases such as cancer, cardiovascular diseases, and autoimmune disorders, which disrupt tissue regulation and impair normal functions (82). Consequently, developing macrophage-targeted drug delivery systems has become a focal point of research. These systems can enhance drug stability, pharmacokinetic properties, controlled release kinetics, and precise temporal drug delivery (83).

In the musculoskeletal system, drug delivery faces numerous challenges. The musculoskeletal system comprises specialized connective tissues such as bones, muscles, cartilage, tendons, and ligaments, which provide protection, structure, mobility, and mechanical performance. However, these tissues experience wear and tear with aging and after injury, necessitating repair (84). Due to the disease burden and treatment demands of the musculoskeletal system, there is an increasing need for regenerative drug therapies targeting musculoskeletal diseases. However, effective drug delivery within the musculoskeletal system remains difficult, posing a significant bottleneck in developing therapeutic drugs for this system (84).

To overcome these challenges, researchers are exploring various innovative strategies. For instance, drug delivery systems developed using nanotechnology can significantly improve drug bioavailability and targeting, showing potential in treating age-related muscle loss (such as sarcopenia) (85). Additionally, stimulus-responsive delivery systems are under continuous development, allowing for precise drug release under specific stimuli, further enhancing therapeutic efficacy (86).

In macrophage-mediated drug delivery systems, researchers are incorporating drugs or drug-loaded nanoparticles into macrophages, macrophage membranes, or macrophage-derived vesicles to extend drug circulation and release time, increase drug stability and targeting ability, and reduce immunogenicity (87). Although these systems have shown potential in treating inflammation, cancer, HIV infection, and other diseases, further research and optimization are needed due to the diversity of their sources and potential differences in their physicochemical properties (87).

In summary, drug delivery research in the macrophage and musculoskeletal diseases field is rapidly advancing, and future research will continue to focus on optimizing these delivery systems to achieve more efficient and precise therapeutic outcomes.

This study has notable limitations inherent to the nature of bibliometrics. First, while the data were sourced from the Web of Science Core Collection (WoSCC), one of the most authoritative databases utilized in bibliometric analyses, certain studies not indexed in WoSCC were excluded. Second, the extraction of all information was performed using bibliometric techniques that rely on machine learning and natural language processing, which could introduce biases not typically found in other bibliometric studies. Nonetheless, we strive to offer scholars a substantial amount of unbiased information and insights.

## 5 Conclusion

This bibliometric study examines the global literature concerning MSK-macrophages. Over the past two decades, there has been a notable increase in research within this area, which is anticipated to continue attracting attention in the foreseeable future. While longitudinal studies and randomized controlled trials are essential for validating existing findings, current research efforts are primarily focused on the development of more precise and targeted therapies. The significant rise in academic publications reflects an expanding interest in the field, suggesting a promising future for this area of study. Global collaboration, particularly spearheaded by institutions in China and the United States, highlights the multinational aspects and broad implications of this research. Notable contributors include Shanghai Jiao Tong University and the University of California. Additionally, journals such as “ARTHRITIS & RHEUMATISM” and “ANNALS OF THE RHEUMATIC DISEASES” serve as vital platforms for advancing this research.

Insights gleaned from keyword exploration, journal relevance, and co-citation analysis reveal critical themes spanning various fields, from immunology to engineering, delineating representative areas for further investigation. A primary limitation of this study is the lack of capacity to manually verify each paper included in the research, which could lead to instances of false reporting. Nonetheless, this bibliometric approach offers researchers, scholars, and students a time-efficient means to identify pertinent research topics and objectives, filter through extensive literature in the field, articulate scientific achievements, and provide a comprehensive overview of the current state of research. Consequently, this study may serve as a valuable guide and reference for researchers contemplating their research directions and choices.
