# Supplementary material for: Unveiling research hotspots: a bibliometric study on macrophages in musculoskeletal diseases
Source: Front Immunol. 2025 Apr 28;16:1519321. doi: 10.3389/fimmu.2025.1519321 (PMC12066445; doi:10.3389/fimmu.2025.1519321)
Supplement: Supplementary file 1 [file DataSheet1.docx]

# Search strategy

| **Search** | **Query** |
| --- | --- |
| #1 | TS=(“macrophage” OR “macrophages” OR “histocyte” OR “histocytes”) |
| #2 | TS=(“Osteoarthritis” OR “Osteoarthritides” OR “Osteoarthrosis” OR “Osteoarthroses” OR “Arthritis Degenerative” OR “Arthritides Degenerative” OR “Degenerative Arthritides” OR “Degenerative Arthritis” OR “Arthrosis, Arthroses” OR “Osteoarthrosis Deformans” OR Osteoporosis OR Osteoporoses OR “Osteoporosis Post-Traumatic” OR “Osteoporosis Post Traumatic” OR “Post-Traumatic Osteoporoses” OR “Post-Traumatic Osteoporosis” OR “Osteoporosis Senile” OR “Osteoporoses Senile” OR “Senile Osteoporoses” OR “Senile Osteoporosis” OR “Osteoporosis Age-Related” OR “Osteoporoses Age-Related” OR “Age-Related Osteoporosis” OR “Age-Related Osteoporoses” OR Osteolysis OR Osteolyses OR “bone infection” OR “bone infections” OR “Arthritis Rheumatoid” OR “Rheumatoid Arthritis” OR osteosarcoma OR Osteosarcomas OR “Osteosarcoma Tumor” OR “Osteosarcoma Tumors” OR “Tumor Osteosarcoma” OR “Tumors Osteosarcoma” OR “Sarcoma Osteogenic” OR “Osteogenic Sarcomas” OR “Sarcomas Osteogenic” OR “Osteogenic Sarcoma” OR “Spinal Cord Injuries” OR “Spinal Cord Trauma” OR “Cord Trauma, Spinal” OR “Cord Traumas, Spinal” OR “Spinal Cord Traumas” OR “Trauma, Spinal Cord” OR “Traumas, Spinal Cord” OR “Myelopathy, Traumatic” OR “Myelopathies, Traumatic” OR “Traumatic Myelopathies” OR “Traumatic Myelopathy” OR “Injuries, Spinal Cord” OR “Cord Injuries, Spinal” OR “Cord Injury, Spinal” OR “Injury, Spinal Cord” OR “Spinal Cord Injury” OR “Spinal Cord Transection” OR “Cord Transection, Spinal” OR “Cord Transections, Spinal” OR “Spinal Cord Transections” OR “Transection, Spinal Cord” OR “Transections, Spinal Cord” OR “Spinal Cord Laceration” OR “Cord Laceration, Spinal” OR “Cord Lacerations, Spinal” OR “Laceration, Spinal Cord” OR “Lacerations, Spinal Cord” OR “Spinal Cord Lacerations” OR “Post-Traumatic Myelopathy” OR “Myelopathies, Post-Traumatic” OR “Myelopathy, Post-Traumatic” OR “Post Traumatic Myelopathy” OR “Post-Traumatic Myelopathies” OR “Spinal Cord Contusion” OR “Contusion, Spinal Cord” OR “Contusions, Spinal Cord” OR “Cord Contusion, Spinal” OR “Cord Contusions, Spinal” OR “Spinal Cord Contusions” OR “cartilage injuries” OR “cartilage injury” OR “bone defects” OR “bone defect” OR “Fractures, Bone” OR “Bone Fracture” OR “Fracture, Bone” OR “Broken Bones” OR “Bone, Broken” OR “Bones, Broken” OR “Broken Bone” OR “Bone Fractures” OR “Spiral Fractures” OR “Fracture, Spiral” OR “Fractures, Spiral” OR “Spiral Fracture” OR “Torsion Fractures” OR “Fracture, Torsion” OR “Fractures, Torsion” OR “Torsion Fracture” OR “Intervertebral Disc Degeneration” OR “Degeneration, Intervertebral Disc” OR “Disc Degeneration, Intervertebral” OR “Intervertebral Disc Degenerations” OR “Disc Degeneration” OR “Degeneration, Disc” OR “Disc Degenerations” OR “Intervertebral Disk Degeneration” OR “Degeneration, Intervertebral Disk” OR “Disk Degeneration, Intervertebral” OR “Intervertebral Disk Degenerations” OR “Disk Degeneration” OR “Degeneration, Disk” OR “Disk Degenerations” OR “Disk Degradation” OR “Degradation, Disk” OR “Disk Degradations” OR “Degenerative Disc Disease” OR “Degenerative Disc Diseases” OR “Disc Disease, Degenerative” OR “Disc Degradation” OR “Degradation, Disc” OR “Disc Degradations” OR “Degenerative Intervertebral Discs” OR “Degenerative Intervertebral Disc” OR “Disc, Degenerative Intervertebral” OR “Intervertebral Disc, Degenerative” OR “Degenerative Intervertebral Disks” OR “Degenerative Intervertebral Disk” OR “Disk, Degenerative Intervertebral” OR “Intervertebral Disk, Degenerative” OR “Arthritis, Gouty” OR “Gouty Arthritis” OR “Arthritides, Gouty” OR “Gouty Arthritides” OR “Spondylitis, Ankylosing” OR “Bechterew's Disease” OR “Bechterews Disease” OR “Marie-Struempell Disease” OR “Marie Struempell Disease” OR “Spondylarthritis Ankylopoietica” OR “Spondyloarthritis Ankylopoietica” OR “Ankylosing Spondylitis” OR “Ankylosing Spondylarthritis” OR “Ankylosing Spondylarthritides” OR “Spondylarthritides, Ankylosing” OR “Spondylarthritis, Ankylosing” OR “Ankylosing Spondyloarthritis” OR “Ankylosing Spondyloarthritides” OR “Spondyloarthritides, Ankylosing” OR “Spondyloarthritis, Ankylosing” OR “Spondylitis Ankylopoietica” OR “Bechterew Disease” OR “Rheumatoid Spondylitis” OR “Spondylitis, Rheumatoid” OR Sarcopenia OR Sarcopenias OR Rhabdomyolysis OR Rhabdomyolyses OR “musculoskeletal diseases” OR “musculoskeletal disease” OR “Orthopedic Disorders” OR “Orthopedic Disorder”) |
| #3 | #1 AND #2 |
